# KART, a knowledge authoring and refinement tool for clinical guidelines development

**DOI:** 10.1186/1753-6561-5-S6-O49

**Published:** 2011-06-29

**Authors:** E Pasche, D Teodoro, J Gobeill, D Vishnyakova, P Ruch, C Lovis

**Affiliations:** 1 Division of Medical Information Sciences, University Hospitals and University of Geneva, Switzerland; 2 Information Science Department, University of Applied Sciences, Geneva, Switzerland

## Introduction / objectives

Optimal antibiotic prescriptions rely on evidence-based clinical guidelines, but creating such guidelines requires a time-consuming systematic review of the literature. We aim at facilitating this process by proposing an innovative tool to extract antibiotic treatments from the literature.

## Methods

We develop a web application, embedding a question-answering (QA) module based on EAGLi (Engine for Question-Answering in Genomics Literature), which has been specifically tuned for antibiotherapy. The users ask questions (i.e. *what antibiotic is used to treat cystitis caused by E. coli?*) to which the system answers by retrieving a set of MEDLINE records from which the most frequently associated antibiotics are extracted and returned in a relevance-ranked list. The users can then access the annotated abstracts of the publications supporting the antibiotic as being a potential treatment, thus allowing them to use their expert judgment to accept or reject the assumption.

## Results

The tool is accessible at http://eagl.unige.ch/KART. The QA engine was able to answer correctly to more than half of the queries (top-precision=0.56). In addition, infectious disease specialists from the University Hospitals of Geneva evaluated KART with several clinical scenarios. Despite an overall appreciation of the system and the recognition of its usefulness, improvements are required to use it when generating or updating clinical practice guidelines.

## Conclusion

KART seeks to facilitate medical knowledge building by providing an advanced retrieval engine. It provides a novel approach to cope with high volumes of literature generated over systematic reviews by facilitating access to pertinent information on antibiotic-related treatments.

## Disclosure of interest

None declared.

